# OncoAID: an open access targeted anti-cancer drugs database

**DOI:** 10.3389/fphar.2025.1588191

**Published:** 2025-08-13

**Authors:** Ohad Landau, Shai Magidi, Catherine Bresson, Fanny Wunder, Razelle Kurzrock, Wafik S. El-Deiry, Kartheeswaran Thangathurai, Eitan Rubin

**Affiliations:** ^1^ The Shraga Segal Department of Microbiology, Immunology and Genetics, Ben-Gurion University of the Negev, Be’er-Sheva, Israel; ^2^ Worldwide Innovative Network (WIN) Association – WIN Consortium, Chevilly-Larue, France; ^3^ Clinical Cancer Center - Froedtert Hospital, Medical College of Wisconsin, Milwaukee, WI, United States; ^4^ Department of Pathology and Laboratory Medicine, Legorreta Cancer Center at Brown University, Providence, RI, United States

**Keywords:** cancer, precision oncology, database, targeted therapy, combinations, artificial intelligence

## Introduction

The rapid evolution of targeted therapies in oncology has revolutionized cancer treatment, offering new hope to patients through precision medicine. These therapies are designed to specifically inhibit molecular targets that drive tumor growth and progression. By focusing on the unique genetic and molecular characteristics of individual tumors, targeted therapies enable more effective and personalized treatment strategies, thereby improving patient outcomes and minimizing adverse effects (Choi and Chang, 2023).

In recent years, the U.S. Food and Drug Administration (FDA) has approved a growing number of targeted therapies, reflecting the increasing understanding of the molecular underpinnings of various cancers. As of 2024, over 150 targeted agents have received FDA approval (Zhong et al., 2021), each associated with specific molecular targets and indications. These therapies encompass a range of mechanisms, including small-molecule inhibitors that disrupt intracellular signaling pathways and monoclonal antibodies that block the interaction between cancer cells and their environment (Zhong et al., 2021; FDA Approved Targeted Drug List, 2024). For instance, agents targeting the epidermal growth factor receptor (EGFR), such as erlotinib and gefitinib, have demonstrated significant efficacy in non-small cell lung cancer (NSCLC) (Maemondo et al., 2010; Toschi et al., 2017), while BRAF inhibitors have transformed the treatment landscape for melanoma (Ribas and Flaherty, 2011; Patel et al., 2020).

To effectively track the landscape of these targeted therapies, comprehensive databases serve as invaluable resources for clinicians and researchers alike. Amid the ongoing artificial intelligent (AI) revolution, such databases hold immense significance for precision oncology algorithms and clinical pharmacology applications, enabling clinicians and researchers to leverage accessible knowledge and tools to make personalized, evidence-based decisions in cancer treatment (Alowais et al., 2023). Comprehensive databases, like PHARMGKB (Whirl-Carrillo et al., 2021), DrugBank (Knox et al., 2024) and Therapeutic Target Database (TTD) (Zhou et al., 2024; Liu et al., 2020; Yang et al., 2024), offer detailed molecular characterization of drugs, pharmacogenomic insights and clinical annotations across numerous therapeutic areas. Clinicians and researchers can use such databases to delve into treatment responses and explore repurposing possibilities. Recently, datasets specifically focusing on anti-cancer drugs (Pantziarka et al., 2021) have been introduced, offering enhanced data availability for machine learning applications and drug repurposing in precision oncology. These resources, though extensive, merely scratch the surface of potential oncology data. The vast possibilities and the comprehensiveness of such databases also introduce challenges, particularly for clinicians who often face time constraints, specifically, when patients fail to respond to guideline-recommended treatments. In such cases oncologists must consider alternative therapeutic approaches by integrating clinical guidelines with available data (Otte et al., 2017; Glatzer et al., 2020).

This report presents the OncoAID database, a specialized and dynamic resource meticulously developed by integration of existing datasets as well as newly curated, up-to-date information. The database is comprehensive, practical, relevant and features an interactive, user-friendly interface that enables clinicians and researchers to quickly and effectively search, filter, and explore information of interest, whether within the database or through navigation to linked external resources.

## Methods

Our criteria to include therapies in the OncoAID database were (i) the treatment was approved by the FDA, and (ii) it is classified as an anti-cancer treatment. Using these criteria, we built the database which catalogs FDA-approved targeted therapies, providing detailed information on each treatment, including names, molecular targets, manufacturers, the specific cancer types for which approvals were granted, approval and withdrawal dates (where applicable), indications for use, efficacy and AI summaries.

**FIGURE 1:**
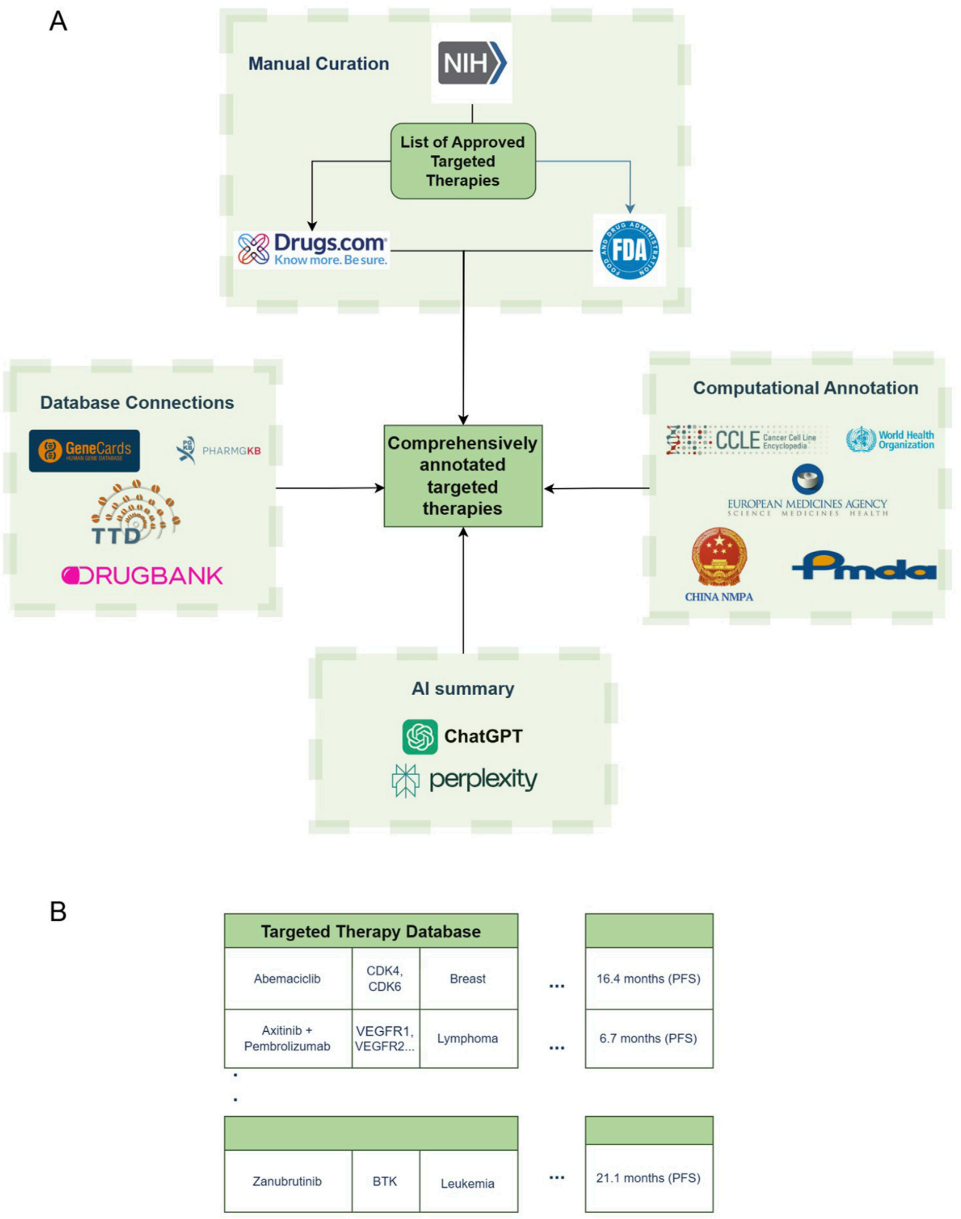
Database creation scheme and building process. **(A)** Data acquisition scheme. **(B)** Sample structure of the database. NIH – National Institutes of Health; FDA – Food and Drug Administration; WHO – World Health Organization; EMA – European Medicines Agency; NMPA – National Medical Products Administration; PMDA – Pharmaceuticals and Medical Devices Agency.

The data collection and processing followed two primary steps: (i) manually compiling a database of targeted anti-cancer therapies, with limited treatment information, and (ii) automating data extraction from other resources, expanding the initial database with relevant information from these resources, as shown in Figure 1.

Initially, FDA-approved targeted anti-cancer therapies, including both single and combination treatments, were gathered from the National Institute of Health (NIH) cancer portal (FDA Approved Targeted Drug List), resulting in a list of 576 distinct treatment regimens covering 31 different cancer types. The NIH portal listed drugs by both their brand names and their international nonproprietary names, which are frequently used in clinical settings. The manufacturing company for each drug was identified through online sources, while details on the drug’s biological target, approval and (if applicable) withdrawal dates, approved uses, cancer types, and specific genomic indications were gathered from sources such as Drugs.com, the NIH and FDA websites, and relevant scientific literature. The database includes URL links to these sources, ensuring traceability of the information provided. This comprehensive manual data gathering and organization led to the creation of a seed database of treatment entries (Figure 1A).

Once curated we obtained progression-free survival (PFS) and overall survival (OS) from FDA and Drugs.com entries when available. We first assembled a corpus with a rigid criteria of information regarding PFS and OS. We then fine-tuned the corpus with a Named Entity Recognition (NER) model from spaCy, an open-source library for NLP in Python. The resulting survival data were incorporated into the dataset as “Survival Rates (FDA)”, Script available upon request. We then leveraged Large Language Models (LLMs) to automatically generate concise summaries for each treatment. We prompted the ChatGPT4o and Perplexity Llama-3.1 models via the application programming interface (API) to provide brief overviews for each of the treatments, setting a 250-word limit for ChatGPT4o and a structured response format for Perplexity. The format includes: 1. “Brief Description and Mechanism”; 2. “PFS and OS Data from Key Clinical Trials”; 3. “Approved Cancer Indications”; and 4. “Additional Notes”. The standardized entries produced by these models are organized under the “ChatGPT4o Says” and “Perplexity Says” columns in the database. It is noteworthy that the LLM-generated responses are concise and intended to be taken as supplementary overview and not for critical decision making.

To enhance drug efficacy assessment, the database integrates data from the Genomics of Drug Sensitivity in Cancer (GDSC) (Yang et al., 2013) and Cancer Cell Line Encyclopedia (CCLE) (Barretina et al., 2012) datasets. The database presents the median IC50 value obtained from these resources (where available) in the ‘IC50’ column. This value, which was derived from multiple cell lines, serves as a broad indicator of treatment’s potency. To further utilize IC50 values as indicators of drug potency, we extracted tissue-specific IC50 data. Each cell line was categorized according to its tissue of origin, and the corresponding IC50 values were used to compute the deviation from the norm (Z-score) for each drug. The resulting distribution of drug sensitivity is represented as a histogram of tissue-specific Z-scores, where data is available.

Additionally, we used the larger Perplexity Llama-large-3.1 model to retrieve the approval statuses for the PMDA in Japan and the NMPA in China for each treatment. Approvals for these regulatory organizations are not readily available and carry language barriers. Therefore, we utilized LLMs, validated and curated the approvals against manually annotated treatment data. Notably, approval statuses from the WHO and EMA were sourced from other existing datasets when available (Pantziarka et al., 2021) and remaining treatments were added through the Llama-large-3.1 LLM model.

In addition, the biological targets of the treatments were linked with GeneCards (Stelzer et al., 2016), enabling users to navigate and explore these targets. The incorporation of other databases, both through external sources and as integrated data extracted directly, is fully automated within our pipeline. This automated process illustrated as the “Database Connections”, “Computational Annotation” and “AI Summary” boxes in Figure 1A, allows for efficient data processing. A sample of the visualized data, following both manual and automated acquisition steps, is shown in Figure 1B.

Once users identify preliminary data of interest within the database, they can access additional connections to comprehensive, well-curated datasets, such as PHARMGKB, DrugBank, TTD, and GeneCards (Whirl-Carrillo et al., 2021; Knox et al., 2024; Zhou et al., 2024; Liu et al., 2020; Yang et al., 2024; Stelzer et al., 2016). Gene targets and treatment identifiers from these external resources were linked directly to the OncoAID interface.

The database interface includes interactive tools that allow users to filter, sort, and extract specific subsets of data for localized exploration. Users can generate downloadable tables, customized views by cancer type, indication, molecular target, and more and can access external curated datasets via embedded hyperlinks. This data can then be easily integrated by researchers into future datasets or AI/ML models, allowing for an in-depth examination that may yield new insights or enhancement of existing knowledge. This structure makes the OncoAID platform not only informative, but also highly actionable for real-world oncology workflows.

## Results

### Database structure

We present here a database of targeted oncology treatments, which includes the following components.

• Drug Name: The international nonproprietary name of the drug.
• Drug Type: Indicates whether the treatment is a single drug or a combination therapy.
• Other Drug Name: Alternate names by which the drug is known, such as brand name.
• Company: The manufacturing company of the drug.
• Target: The primary target of the drug.
• Genomics: Genomic information pertaining to the drug’s approval.
• Indication: Specific indications for which the drug is used.
• Cancer Type: The primary type of cancer the drug addresses.
• Reference: Source of manually curated data, such as Drugs.com or the FDA website.
• Approval Date: The date the drug received FDA approval.
• Comments: Clinical notes related to the approval of the drug.
• Withdrawal Date: The date when FDA approval for the drug was withdrawn, if applicable.
• PharmGKB, DrugBank, TTD: Clickable links to the drug’s PharmGKB, DrugBank and TTD datasets.
• Survival Rates (FDA): Survival rates extracted from FDA references using in-house named entity recognition (NER).
• ChatGPT4 Says/Perplexity Says: Brief summaries generated by AI models.
• FDA/EMA/WHO/PMDA*/NMPA*: Approval status according to various international regulatory agencies. ‘*’ indicates data was partially or fully sourced from AI tools.
• IC50µM/IC50 Tissue Specific (Z Score): Data on cell line drug sensitivity.
• Survival Rates (FDA)/Chat GPT4o Says/Perplexity Says PFS/OS (Months): When available, columns consisting of the median PFS/OS supplied by the data source, in months.

We also present an interface that utilizes this structured database to allow further querying and filtering tailored to clinicians’ needs. This interface was designed to reduce browsing complexity and facilitating the efficient selection of actionable drugs for deeper exploration within comprehensive datasets.

### Database statistics

As of November 2024, the OncoAID curated dataset encompassed information on 576 regimens, with 303 unique treatments (164 anti-cancer drugs and 139 anti-cancer drug combinations), covering a total of 345 distinct indications. The dataset provides median PFS for 407 and OS data for 288 entries, both single agents and combinations. IC50 values are available for 87 entries, as provided by the Genomics of Drug Sensitivity in Cancer (GDSC). All entries are accompanied by concise AI LLMs generated summaries.

To demonstrate the utility of the database, we used it to answer several questions: (1) is there a trend in approval rate over time? (2) do different approval agencies differ in the treatments they approve? (3) do combinations really benefit the patients?

**FIGURE 2:**
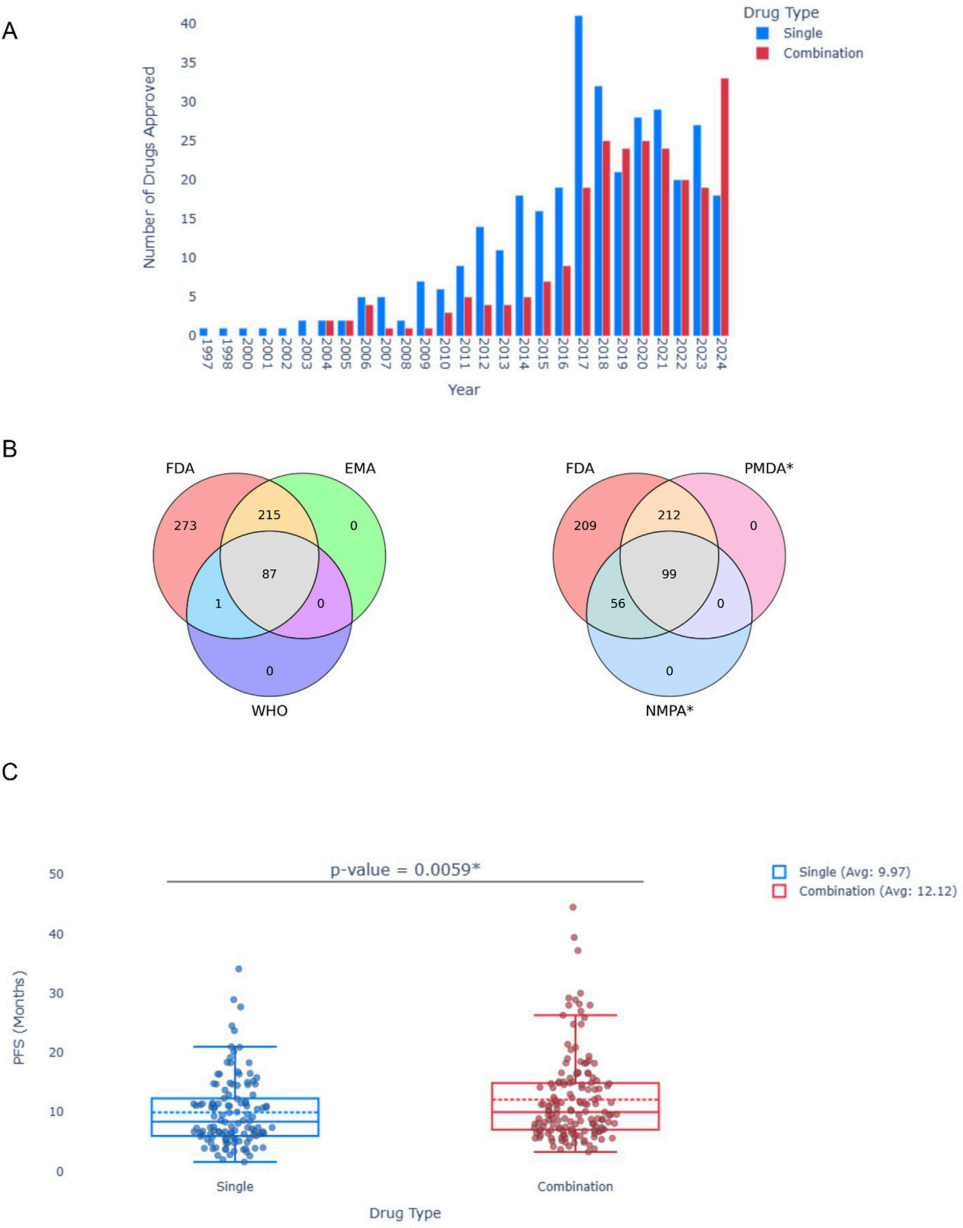
Statistical Analysis of the OncoAID database. **(A)** Approval of Single or Drug combination per year. **(B)** Venn diagrams of approvals per organization (FDA, EMA and WHO–Left; FDA, PMDA^*^ and NMPA^*^ Right). **(C)** Box plot of median PFS across all single and drug combinations in the data. Mann-Whitney u test; p value = 0.0059.

For (1), we used the database to create a histogram illustrating the number of approvals per year (Figure 2A). A marked increase in the approval of targeted cancer therapies is obvious, all the way to 2024, with a notable surge in drug combinations over the past decade. For (2), a Venn Diagram (Figure 2B) depicts the divergence in approvals among different organizations. It is important to note that, under our inclusion criteria, only FDA-approved drugs were considered; thus, all entries fall within the FDA circle. However, authorizations from WHO, EMA, NMPA and PMDA were obtained through the database, allowing for a comparative visualization of the differing approaches taken by these organizations. Lastly, for (3), a Box Plot comparing the median PFS in months of 407 single agents and combinations was created (Figure 2C). Notably, the average median PFS for drug combinations was significantly greater for combinations (12.12 compared to 9.97 months; p-value = 0.0059, Mann-Whitney u test). This preliminary statistical analysis demonstrates the potential value of OncoAID as a tool for addressing both clinical and research inquiries in oncology.

## Discussion

The OncoAID curated database represents a significant advancement in the landscape of cancer treatment resources, offering clinicians and researchers a comprehensive, open-access database and an interface for navigating FDA-approved anti-cancer targeted therapies as single agent or combinations. The database integrates a variety of efficacy data, including PFS and OS statistics from clinical trials, as well as IC50 values from cell line studies. By providing detailed information on drug efficacy, molecular targets, approval statuses, and genomic markers, the database empowers personalized treatment approaches, crucial for advancing precision oncology. The interface further provides powerful visualizations and search capabilities.

One of the key strengths of the database lies in its integration of both clinical trial outcomes and laboratory-based studies. To our knowledge, it is the first open-access database designed for this purpose, incorporating comprehensive data on anti-cancer targeted treatments with their associated efficacy, both from clinical trials (PFS and/or OS) and from cell line studies. This dual approach—combining real-world clinical data with experimental findings enhances the utility of the database for both clinical decision-making and translational research.

In addition, the incorporation of AI-generated summaries from models like ChatGPT4o and Perplexity Llama-large-3.1 further streamlines the process of accessing relevant information, making the tool accessible for time-constrained clinicians while maintaining scientific rigor.

The core novelty of OncoAID lies in the integration of curated clinical efficacy data, regulatory insights, and interactive navigation features into a unified precision oncology tool. While the use of AI models in this project does not aim to introduce methodological innovation, it serves a practical by enhancing content summarization and usability.

The database also stands out in its global scope, incorporating approvals not only from the FDA but also from the EMA, WHO, and other regulatory bodies, such as the PMDA in Japan and the NMPA in China. This comprehensive approach allows users to understand the varying regulatory landscapes and the global application of treatments, which is essential for clinicians working in diverse healthcare environments. Notably, large gaps make collecting this information difficult. Language barriers and limited access to approval data from certain regions required innovative methods utilizing large language models to bridge these gaps. The global scope is extremely valuable in some settings. In fact, this is essential for the WIN Consortium, which holds International Molecular Tumor Boards discussing cases from various sites around the world with a panel of experts in precision oncology. Such a tool can be a valuable resource for the panel of experts discussing the presented patients. Future versions of the database may include the approval status of treatments by additional international regulatory agencies and may incorporate treatments that are approved outside of the FDA, enhancing its global utility. The OncoAID database is not without limitations. Using FDA approval as an inclusion criterion means that treatments authorized only by other regulatory bodies (even if FDA approval will be granted at a later date) may be missed. This could be a significant limitation and may even become critical in certain global contexts. Another limitation is the integration of AI-generated summaries. These summaries may lack the depth required for highly specialized clinical decisions, may be incomplete and may even include false information, also known as “hallucinatuins” (Sun et al., 2024). As such, they should be interpreted with caution and used as supplementary tools rather than sole sources of information. Finally, the reliance on AI models for data generation and processing leads to uncertainties regarding accuracy. Despite implementing a rigorous validation process, there is always the possibility of errors or inconsistencies in the automatically generated data. As AI technologies evolve, continuous improvements will be performed and as additional layers of validation will be performed, any uncertainty regarding accuracy will decrease.

Unlike existing resources which provide specific annotations (e.g., focus on pharmacogenomics and chemical properties), the OncoAID database offers integrated annotations tailored for precision oncology, covering beyond single treatments. The annotations include AI-generated treatment summaries, curated PFS/OS data, integration of drug sensitivity metrics (IC50 and tissue-specific Z-scores), approval status across multiple international regulatory bodies (e.g., FDA, EMA, PMDA, NMPA), and interactive filtering tools designed for clinical applicability. These combined features make OncoAID a practical, clinician-focused tool that combines clinical trial data, molecular pharmacology, and global regulatory status in a single, user-friendly interface. To the best of our knowledge, our database adheres to the FAIR principles to ensure Findability, Accessibility, Interoperability, and Reusability. It features unique identifiers and detailed metadata to support data discovery and citation. The database is designed for easy access with clear usage policies, while standardized authentication methods can be applied in case restricted access is mandated. Interoperability is achieved through the use of common data standards and controlled vocabularies, promoting seamless integration with other datasets. For Reusability, we provide an open license, documentation, version control, and transparent data provenance to encourage responsible use. We also include example queries and analysis code to assist users in making the most of the data.

## Conclusion

In conclusion, the OncoAID database represents a pivotal resource in the field of precision oncology. It merges cutting-edge AI capabilities with comprehensive, curated data to support informed decision-making in cancer treatment selection. By providing succinct yet comprehensive information about targeted treatment options including combinations and by enabling access to both clinical and experimental data, the database provides a valuable tool for clinicians and researchers. Ongoing efforts to broaden its scope, to further improve the accuracy of AI-generated data and to address the limitations discussed above, will aim at improving its utility and making the database even more useful. These improvements, as well as constant updates, will ensure its utility in the fast-evolving landscape of cancer therapeutics.

Access to the database is available at https://fohs.bgu.ac.il/rubinlab/oncoaid/through the interface we describe here.

## Data Availability

The datasets presented in this study can be found in online repositories. The names of the repository/repositories and accession number(s) can be found below: https://fohs.bgu.ac.il/rubinlab/oncoaid/.

## References

[B1] Alowais S. A. Alghamdi S. S. Alsuhebany N. Alqahtani T. Alshaya A. I. Almohareb S. N. (2023). Revolutionizing healthcare: the role of artificial intelligence in clinical practice. BMC Med. Educ. 23, 689. 10.1186/s12909-023-04698-z 37740191 PMC10517477

[B2] Barretina J. Caponigro G. Stransky N. Venkatesan K. Margolin A. A. Kim S. (2012). The Cancer Cell Line Encyclopedia enables predictive modelling of anticancer drug sensitivity. Nature 483, 603–607. 10.1038/nature11003 22460905 PMC3320027

[B3] Choi H. Y. Chang J. E. (2023). Targeted therapy for cancers: from ongoing clinical trials to FDA-approved drugs. Int. J. Mol. Sci. 24, 13618. 10.3390/ijms241713618 37686423 PMC10487969

[B5] FDA Approved Targeted Drug List (2024). List of targeted therapy drugs approved for specific types of cancer. Available online at: https://www.cancer.gov/about-cancer/treatment/types/targeted-therapies/approved-drug-list (Accessed November 11, 2024).

[B6] Glatzer M. Panje C. M. Sirén C. Cihoric N. Putora P. M. (2020). Decision making criteria in oncology. Oncol. Switz. 98, 370–378. 10.1159/000492272 30227426

[B7] Knox C. Wilson M. Klinger C. M. Franklin M. Oler E. Wilson A. (2024). DrugBank 6.0: the DrugBank Knowledgebase for 2024. Nucleic Acids Res. 52, D1265–D1275. 10.1093/nar/gkad976 37953279 PMC10767804

[B8] Liu H. Zhang W. Zou B. Wang J. Deng Y. Deng L. (2020). DrugCombDB: a comprehensive database of drug combinations toward the discovery of combinatorial therapy. Nucleic Acids Res. 48, D871–D881. 10.1093/nar/gkz1007 31665429 PMC7145671

[B9] Maemondo M. Inoue A. Kobayashi K. Sugawara S. Oizumi S. Gemma A. (2010). Gefitinib or chemotherapy for non-small-cell lung cancer with mutated EGFR. N. Engl. J. Med. 362, 2380–2388. 10.1056/NEJMoa0909530 20573926

[B10] Otte I. Salloch S. Reinacher-Schick A. Vollmann J. (2017). Treatment recommendations within the leeway of clinical guidelines: a qualitative interview study on oncologists’ clinical deliberation. BMC Cancer 17, 780. 10.1186/s12885-017-3783-6 29162047 PMC5699200

[B11] Pantziarka P. Capistrano I. R. De Potter A. Vandeborne L. Bouche G. (2021). An open access database of licensed cancer drugs. Front. Pharmacol. 12. 10.3389/fphar.2021.627574 PMC7991999 33776770

[B12] Patel H. Yacoub N. Mishra R. White A. Long Y. Alanazi S. (2020). Current advances in the treatment of braf-mutant melanoma. Cancers 12, 482. 10.3390/cancers12020482 32092958 PMC7072236

[B13] Ribas A. Flaherty K. T. (2011). BRAF targeted therapy changes the treatment paradigm in melanoma. Nat. Rev. Clin. Oncol. 8, 426–433. 10.1038/nrclinonc.2011.69 21606968

[B14] Stelzer G. Rosen N. Plaschkes I. Zimmerman S. Twik M. Fishilevich S. (2016). The GeneCards suite: from Gene data mining to Disease Genome Sequence Analyses. Curr. Protoc. Bioinforma. 54 (1), 1.30.1–1.30.33. 10.1002/cpbi.5 27322403

[B15] Sun Y. Sheng D. Zhou Z. Wu Y. (2024). AI hallucination: towards a comprehensive classification of distorted information in artificial intelligence-generated content. Humanit Soc. Sci. Commun. 11, 1278. 10.1057/s41599-024-03811-x

[B16] Toschi L. Rossi S. Finocchiaro G. Santoro A. (2017). Non-small cell lung cancer treatment (r)evolution: Ten years of advances and more to come. ecancermedicalscience 11, 787. 10.3332/ecancer.2017.787 29225694 PMC5718252

[B17] Whirl-Carrillo M. Huddart R. Gong L. Sangkuhl K. Thorn C. F. Whaley R. (2021). An evidence-based Framework for evaluating pharmacogenomics knowledge for personalized medicine. Clin. Pharmacol. Ther. 110, 563–572. 10.1002/cpt.2350 34216021 PMC8457105

[B18] Yang J. Zhuang X. Li Z. Xiong G. Xu P. Ling Y. (2024). CPMKG: a condition-based knowledge graph for precision medicine. Database. 2024. Oxford. 10.1093/database/baae102 PMC11429523 39331730

[B19] Yang W. Soares J. Greninger P. Edelman E. J. Lightfoot H. Forbes S. (2013). Genomics of Drug Sensitivity in Cancer (GDSC): a resource for therapeutic biomarker discovery in cancer cells. Nucleic Acids Res. 41, D955–D961. 10.1093/nar/gks1111 23180760 PMC3531057

[B20] Zhong L. Li Y. Xiong L. Wang W. Wu M. Yuan T. (2021). Small molecules in targeted cancer therapy: advances, challenges, and future perspectives. Signal Transduct. Target. Ther. 6, 201. 10.1038/s41392-021-00572-w 34054126 PMC8165101

[B21] Zhou Y. Zhang Y. Zhao D. Yu X. Shen X. Zhou Y. (2024). TTD: *Therapeutic Target Database* describing target drug ability information. Nucleic Acids Res. 52 (D1), D1465–D1477. 10.1093/nar/gkad751 37713619 PMC10767903

